# The Electro-Magnet in Eye Work—Its Use in Two Cases

**Published:** 1905-03

**Authors:** Albert C. Snell

**Affiliations:** Rochester, N. Y.


### The Electro-magnet in Eye Work—Its Use in Two Cases.

By ALBERT C. SNELL, M. D., Rochester, N. Y.

CASE I.—On August 5, 1904, Mr. C., 26 years of age, was sent to me by Dr. Percy with the statement that the patient had been struck in his left eye by a piece of steel. Examination showed a clean-cut vertical wound of the cornea, 2 m.m. long, situated opposite the nasal edge of the dilated pupil; a small nick in the iris; and an opaque and swollen lens. Vision was reduced to light perception only. With the ophthalmoscope. no part of the fundus of the eye could be seen. The foreign body had apparently passed through cornea, iris and lens and had lodged somewhere in the vitreous or deeper.

In order to better determine the presence of a foreign body, Dr. Greenleaf was requested to make an *x*-ray picture. The plate showed the presence of the steel and indicated that it was deeply situated.

Patient was sent to the City Hospital and under a general anesthetic, the cornea was incised, an iridectomy performed, and the tip of a small electro-magnet introduced in the wound. After making repeated attempts with the various tips to find the steel and failing to attach it, the operation was desisted in.

Two days later the eye developed all the symptoms of panophthalmitis and the patient then consented to have the eye enucleated. This was done and the eye immediately opened. A triangular sliver of steel, 3 m.m. long and 1 m.m. in its thickest part, was found lying to the nasal side of the optic disc. It was imbedded in the sclera and covered with lymph and pus. The reason for failure to extract the steel was evidently due to its attachment in the sclera. And even had it been successfully removed the eye-ball would have been lost from infection; as the steel was from the edge of a dirty, greasy tool and at the time of the magnet operation evidences of infection were found.

Case II.—Peter N., age 35, was struck in the left eye, June 7, 1904, by a piece of iron while breaking up scrap iron with a sledge hammer. Two years previously he had had a similar accident to his right eye which resulted in complete blindness of that eye. Examination of the left showed an open clean-cut wound, 4 m.m. long extending obliquely across the center of the cornea; the sphincter of the iris slightly cut down and in; and the lens cataractous. Vision was light perception.

The patient was sent to the wards of the City Hospital where Dr. Andrew made an *x*-ray picture, which showed the presence of the foreign body. Under a general anesthetic the electro-magnet operation was performed. Forty-eight hours had elapsed since the accident and the wound was closed, therefore an incision

---

1. Read at the 37th annual meeting of the Medical Association of Central New York, held at Rochester, N. Y., October 18, 1904.

was made above through the cornea similar to the incision for cataract. An iridectomy was done and the greater part of the soft lens removed by pressure and curette. The iron was not found in the lens. The short, blunt tip of the magnet was then introduced to the depth of the posterior surface of the lens and on withdrawing it there was found attached to it three small pieces of metal, ½ m.m. or less in their largest dimension. These were demonstrated to be metal by again magnetising them after wiping them from the tip. A longer and curved tip was now introduced and slowly pushed back to about one half the depth of the vitreous when a decided click was heard and the tip on being withdrawn was seen to have a piece of metal attached to it. This was seized between the lips of the wound with a pair of forceps and removed. It is a thin egg-shaped piece 7 m.m. long and 3 m.m. broad.

The wound healed promptly and the eye made a good recovery. With a + 10 sph. lens patient has $\frac{20}{40}$ vision which enables him to follow a useful occupation and to earn his livelihood. He was last seen October 4 and his excellent vision was still maintained.

I report these two cases not because of novelty either in the cases themselves or in the use of the electro-magnet. They are examples of a failure and of a success and are illustrative of a large number of similar cases which have been reported at various times. The use of the electro-magnet for removal of metal from the eye was first employed by Hirschberg and by Dr. Bradford in 1881. The permanent magnet was used previous to this date but was found to be of little value on account of its weak pulling power. Since this date the electro-magnet has been made in various sizes from the original Hirschberg, which weighs about a pound and is easily handled, to the Haab giant magnet which is non-portable on account of its size and weight and has pulling strength enough to pick pockets of watches.

Successful recovery of an eye which has had metal removed from it depends largely on two factors, infection and location. The nearer the metal lodges to the anterior surface of the eye the less the danger of destructive infection and the less the danger of doing irreparable damage to vision in the removal. The deeper it lodges the greater these dangers. A large percentage of foreign bodies lodged in the iris or lens are removed successfully both as to saving the eye-ball and as to saving its vision, while useful vision following extraction of metal from the vitreous is the exception.

My second case presents a point of special interest: the fact that with a single wound of entrance there existed within the eye-ball several pieces of metal lying in different locations. This demonstrates that a flying piece of metal in passing through the cornea or the lens may be broken in two or more fragments. And, as in this case, some fragments may be deposited along its path while the main piece may lodge deeper.

Dr. Wheelock Rider reported in 1899 to the American Ophthalmological Society (Vol. viii., p. 574,) a case in which, after the eye had been enucleated there were found three pieces of steel, one in the lens, one in ciliary body, and one in the fundus. But, so far as I have been able to find out, no case has been described in which, before enucleation, there has been extracted from the eye more than one piece of metal having a single wound of entrance.

53 South Fitzhugh Street.

